# Oxygen-dependent bond formation with FIH regulates the activity of the client protein OTUB1

**DOI:** 10.1016/j.redox.2019.101265

**Published:** 2019-07-02

**Authors:** Christina Pickel, Julia Günter, Amalia Ruiz-Serrano, Patrick Spielmann, Jacqueline-Alba Fabrizio, Witold Wolski, Daniel J. Peet, Roland H. Wenger, Carsten C. Scholz

**Affiliations:** a Institute of Physiology, University of Zurich, 8057, Zurich, Switzerland; b National Centre of Competence in Research ‘Kidney.CH’, Switzerland; c School of Biological Sciences, University of Adelaide, Adelaide, SA, 5005, Australia; d Functional Genomics Center Zurich, University of Zurich, 8057, Zurich, Switzerland

**Keywords:** Hydroxylase, HIF, Hypoxia, Oxygen sensor, Deubiquitinase, Ubiquitin system

## Abstract

Protein:protein interactions are the basis of molecular communication and are usually of transient non-covalent nature, while covalent interactions other than ubiquitination are rare. For cellular adaptations, the cellular oxygen and peroxide sensor factor inhibiting HIF (FIH) confers oxygen and oxidant stress sensitivity to the hypoxia inducible factor (HIF) by asparagine hydroxylation. We investigated whether FIH contributes to hypoxia adaptation also through other mechanisms and identified a hypoxia sensitive, likely covalent, bond formation by FIH with several client proteins, including the deubiquitinase ovarian tumor domain containing ubiquitin aldehyde binding protein 1 (OTUB1). Biochemical analyses were consistent with a co-translational amide bond formation between FIH and OTUB1, occurring within mammalian and bacterial cells but not between separately purified proteins. Bond formation is catalysed by FIH and highly dependent on oxygen availability in the cellular microenvironment. Within cells, a heterotrimeric complex is formed, consisting of two FIH and one covalently linked OTUB1. Complexation of OTUB1 by FIH regulates OTUB1 deubiquitinase activity. Our findings reveal an alternative mechanism for hypoxia adaptation with remarkably high oxygen sensitivity, mediated through covalent protein-protein interactions catalysed by an asparagine modifying dioxygenase.

## Highlights

• FIH forms a (likely amide) bond with client proteins.
• Bond formation is highly hypoxia sensitive and occurs co-translationally.
• FIH forms a heterotrimer with the client protein OTUB1 (FIH_2_OTUB1_1_).
• Complex formation between OTUB1 and FIH regulates OTUB1 deubiquitinase activity.
• Bond formation by hydroxylases is an alternative mechanism for hypoxia adaptation.

## Graphical abstract

**Figure:**
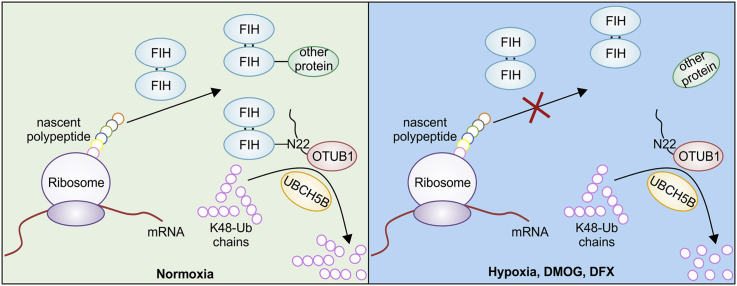

## 1 Introduction

Cellular oxygen sensing is of vital importance for cells and tissues in order to adapt to hypoxic conditions when cellular oxygen demand exceeds its supply [1]. The best characterized cellular oxygen sensors are the prolyl-4-hydroxylase domain (PHD) proteins 1–3 and factor inhibiting HIF (FIH) [2]. PHDs hydroxylate two different prolines and FIH hydroxylates one asparagine residue of HIFα subunits [2]. Besides molecular oxygen, these enzymes require Fe^2+^ and ascorbate or other reducing agents as co-factors and 2-oxoglutarate as co-substrate in order to reduce molecular oxygen and oxidize the substrate protein (hydroxylation) and 2-oxoglutarate (conversion to succinate) [3,4]. Proline-4-hydroxylation of HIFα leads to its proteasomal degradation whereas asparagine hydroxylation inhibits its interaction with the transcriptional co-activators p300 and CBP, attenuating HIF-dependent gene transactivation [2]. While in higher organisms the only known reaction of 2-oxoglutarate-dependent dioxygenases is hydroxylation [5], in lower organisms they also catalyse ring expansion, rearrangement, desaturation, halogenation and epoxidation [6].

Beside oxygen, FIH also senses peroxide [7]. Interestingly, FIH is more sensitive to H_2_O_2_ than the PHDs [7]. Peroxide reduces FIH enzymatic activity, leading to decreased HIF-1α asparagine hydroxylation and higher transcriptional activity [7]. This indicates that FIH functionally integrates oxidant stress and hypoxia in cellular signaling.

*In vivo*, FIH is essential for the regulation of energy metabolism [8,9]. Amongst others, FIH deletion leads to an increased metabolic rate, increased glucose and lipid homeostasis and increased oxidative metabolism [8,9]. The FIH-dependent regulation of energy metabolism is at least partly independent of HIF [8]. Therefore, a key question remaining is whether FIH regulates additional substrates outside of the HIF pathway that contribute to the observed phenotype. FIH has previously been shown to target proteins for hydroxylation other than HIFα, including ankyrin repeat domain (ARD)-containing proteins [[10], [11], [12], [13], [14], [15]]. However, whether FIH-dependent hydroxylation of these proteins is functionally relevant for the regulation of energy metabolism is unclear [11,16]. We recently demonstrated that FIH interacts with the deubiquitinase (DUB) ovarian tumor domain containing ubiquitin aldehyde binding protein 1 (OTUB1) and hydroxylates it on asparagine 22 (N22), regulating cellular energy metabolism [17,18].

OTUB1 is a ubiquitously expressed DUB with one of the highest expression levels of all DUBs [19,20]. OTUB1 cleaves K48-ubiquitin chains through its canonical enzymatic activity, preventing proteasomal degradation of substrate proteins [21,22]. In addition, OTUB1 inhibits E2 ubiquitin-conjugating enzymes independent of its enzymatic activity, impeding ubiquitin chain formation [[23], [24], [25]]. OTUB1 enzymatic activity is regulated by complexation with E2 enzymes and free ubiquitin [26,27]. A major characteristic of DUBs is their frequent occurrence in protein complexes, which controls DUB activity [28]. However, whether other proteins in addition to E2 enzymes and free ubiquitin affect OTUB1 activity by protein complexation is unknown.

In this study, we further investigated the molecular interplay between FIH and OTUB1. We show evidence for an unexpected formation of a previously unknown strong, likely covalent, interaction between FIH and OTUB1. We demonstrate that this formation has functional consequences for OTUB1 deubiquitinase activity and is highly oxygen sensitive but relatively slow, indicating a role in chronic hypoxia adaptation. Interestingly, this type of FIH-dependent bond formation is likely not restricted to the substrate OTUB1.

## 2 Materials and methods

### 2.1 Cell culture and transient transfection

Human HEK293 (embryonic kidney), MCF7 (breast adenocarcinoma) and Hep3B (hepatocellular carcinoma) cell lines were cultured in DMEM containing 4.5 g/l glucose, sodium pyruvate and l-glutamine (Sigma-Aldrich, St Louis, MO, USA), supplemented with 10% heat-inactivated fetal bovine serum (Gibco by Life Technologies, Carlsbad, Ca, USA) and 100 U/ml penicillin and 100 μg/ml streptomycin (Sigma-Aldrich). Transient transfection of siRNAs and plasmids was performed using lipofectamine 2000 reagent according to the manufacturer's instructions (Invitrogen, Carlsbad, CA, USA) or polyethylenimine (PEI; Polysciences Inc., Warrington, PA) as described previously [29].

### 2.2 Cell treatments

Cycloheximide (CHX; Sigma-Aldrich) was dissolved in ethanol; desferrioxamine (DFX; Sigma-Aldrich), (+)-sodium l-ascorbate (Sigma-Aldrich), 2-oxoglutarate (Sigma-Aldrich), diethyl 2-oxoglutarate (DE-2OG; Sigma-Aldrich) and iron (II) sulfate (Sigma-Aldrich) in H_2_O; and dimethyloxalylglycine (DMOG; Frontier Scientific, Logan, UT, USA) and FG-4592 (Roxadustat; Selleckchem, Houston, TX, USA) in dimethylsulfoxide (DMSO, Sigma-Aldrich). Hypoxic incubations were performed using the InvivO_2_ 400 humidified cell culture workstation (Baker Ruskinn, Bridgend, South Wales, UK) operated at 0.2% O_2_ and 5% CO_2_ as described previously [29] or in humidified oxygen-regulated cell culture incubators (Binder GmbH, Tuttlingen, Germany) operated at 1%–8% O_2_ and 5% CO_2_. If not otherwise indicated, “normoxia” refers to the standard oxygen concentration in the gas phase within a cell culture incubator at 500 m altitude (18.5% O_2_) and “hypoxia” to 0.2% O_2_ [30].

### 2.3 Plasmids and siRNAs

The plasmid encoding human wildtype FIH-V5 was kindly provided by Dr. Eric Metzen (University of Duisburg-Essen, Essen, Germany). The plasmids encoding FIH H199A-V5 and MBP-FIH have previously been described [31]. The plasmid coding for human wildtype FLAG-OTUB1 (containing two consecutive FLAG tags) [32] was a kind gift of Dr. Mu-Shui Dai (Oregon Health & Science University, Portland, OR, USA). The transfer vector pET3a and the polycistronic expression vector pST39 [33] were kind gifts from Prof. Song Tan (Pennsylvania State University, PA, USA).

Nontargeting siRNA (siNT: 5′-gcuccggagaacuaccagaguauua-3′) as well as siRNA targeting human FIH (siFIH: sequence F1, 5′-guugcgcaguuauagcuuctt-3′) and the 3′UTR of human OTUB1 (siOTUB1: siRNA-4, 5′-gugguuguaaaugguccuatt-3′) were purchased from Microsynth (Balgrach, Switzerland) according to previously reported sequences [10,34].

### 2.4 Generation of OTUB1 mutants by site-directed mutagenesis

The human FLAG-OTUB1 N22A mutant was previously described [18]. The human FLAG-OTUB1 S16A, S18A, S16A/S18A, C23A, C23S, C91A, C91S point mutants were generated using the Quikchange II XL Site-Directed Mutagenesis kit (Agilent Technologies, Santa Clara, CA, USA) according to the manufacturer's instructions and using the plasmid encoding wildtype FLAG-OTUB1 as template. The mutations of the target sites were confirmed by sequencing.

### 2.5 Immunoblot analysis

Cells were lysed in 150 mM NaCl, 25 mM Tris-HCl (pH 8.0), 1 mM EDTA, 1% NP-40, freshly supplemented with protease inhibitor cocktail (Sigma-Aldrich), 1 mM PMSF and 1 mM Na_3_VO_4_ and 100 mM iodoacetamide where indicated. Protein concentrations of lysates were determined using the BCA assay (Thermo Fisher Scientific, Waltham, MA, USA). Equal amounts of proteins were mixed with 5x loading dye (250 mM Tris-HCl pH 6.8, 30% glycerol, 858 mM β-mercaptoethanol, 10% SDS, 0.05% bromophenolblue), separated by SDS-PAGE, electro-transferred to nitrocellulose membranes and detected using anti-FIH antibody (Novus Biologicals, Littleton CO, USA; NBP1-30333), anti-V5 antibody (Invitrogen; R960-025), anti-OTUB1 antibody (Cell Signaling Technology, Danvers, MA, USA; 3783), anti-FLAG antibody (Sigma-Aldrich; F3165), anti-ubiquitin antibody (clone P4D1; Cell Signaling Technology; 3936), anti–HIF–1α antibody (BD Biosciences, San Jose, CA, USA; 610959), anti–HIF–2α antibody (Bethyl Laboratories, Montgomery, TX, USA; A700-003), anti-GFP antibody (Roche Diagnostics, Rotkreuz, Switzerland; 11814460001), anti-β-actin antibody (Sigma-Aldrich; A5441), anti-α-tubulin antibody (Cell Signaling; 2144), anti-SMC1 antibody (Abcam, Cambridge, UK; 9262) and horseradish peroxidase-coupled secondary antibodies (Thermo Fisher Scientific; 31430, 31460). Bound antibodies were detected with SuperSignal enhanced chemiluminescence substrate (Thermo Fisher Scientific) and chemiluminescence was recorded using a CCD camera (LAS 4000 mini, Fujifilm, Tokyo, Japan). ImageQuant TL gel analysis software (GE Healthcare, Version 8.1) was used for quantification as previously described [35]. If not indicated otherwise, values were normalized to the respective loading control and the sum of the intensities of all samples of one signal of each experiment.

### 2.6 Denaturing urea and blue native electrophoresis

For urea electrophoresis, cells were harvested in 30 mM Tris-HCl (pH 8.5), 7 M urea, 2 M thiourea, 0.4% CHAPS, supplemented with protease inhibitor cocktail (Sigma-Aldrich) as described [36]. The protein concentration was determined by Bradford assay and equal protein amounts were separated by 8% urea gel electrophoresis according to the previous description [37,38]. For blue native electrophoresis, cells were harvested in 20 mM Bis-Tris (pH 7.0), 500 mM ε-aminocaproic acid, 10% glycerol, supplemented with 1 mM PMSF, 10 μg/ml aprotinin, 10 μg/ml leupeptin and 1 mM Na_3_VO_4_, and lysed by dounce homogenisation. The protein concentration was determined by Bradford assay and equal protein amounts were separated by 15% blue native gel electrophoresis according to a previous description [39]. Following electrophoresis, proteins were transferred to nitrocellulose membranes and detected using antibodies as described above.

### 2.7 Two-dimensional gel electrophoresis

Cell lysates were prepared for native protein analysis and separated by blue native electrophoresis as described above. Single lanes were cut and separated in the second dimension by SDS-PAGE in 100 mM Tris-HCl (pH 6.8), 12% glycerol, 343 mM β-mercaptoethanol, 4% SDS, 0.02% bromophenolblue [40]. Following transfer to nitrocellulose membranes, proteins were detected using antibodies as described above.

### 2.8 Bacterial expression and purification of His- and MBP-tagged recombinant proteins

The plasmids encoding human pENTR4-OTUB1 WT/N22A were described previously [18] and utilized for generating pDEST17-OTUB1 WT/N22A (coding for His-OTUB1 WT/N22A) using the Gateway system according to the manufacturer's description (Invitrogen). His-OTUB1 WT/N22A was subcloned into the bacterial expression vector pET3a using pDEST17-OTUB1 WT/N22A plasmids as templates. For cloning of MBP-tagged human FIH into pET3a, FIH WT/H199A was subcloned using pFIH WT/H199A-V5 as template, followed by subcloning of an N-terminal MBP tag using human pMBP-FIH as template. *E.coli* BL21(DE3)pLysS (Invitrogen) were transformed with the plasmids and expression of the respective proteins was induced by addition of 0.2 mM isopropyl-ß-D-thiogalactoside (IPTG) for up to 6 h at 30°C. For purification of His-tagged proteins, bacteria were resuspended in 20 mM Tris-HCl (pH 8.0), 500 mM NaCl, 5 mM imidazole, and for purification of MBP-tagged proteins in 20 mM Tris-HCl (pH 8.0), 150 mM NaCl. Lysis buffers were supplemented with 1 mM PMSF and protease inhibitor cocktail (Sigma-Aldrich). Bacteria were lysed using a cell disruptor (TS Series Bench Top, Constant Systems Ltd., Northants, UK) in two cycles at 35 kPsi. Lysates were cleared by ultracentrifugation at 162,000 g, 4°C for 1 h and proteins were affinity purified with NiSO_4_-charged sepharose (HiTrap Chelating HP, GE Healthcare, Little Chalfot, UK) or dextrin sepharose (MBPTrap HP, GE Healthcare 28-918-780) columns using the Duo Flow system (Bio-Rad, Hercules, CA, USA). Protein concentrations were determined by Bradford assay. Dot blot, colloidal Coomassie staining [41] and OTUB1 and FIH immunoblotting were used to verify successful protein expression and purification.

### 2.9 Bacterial expression and purification of the stable FIH-OTUB1 complex from a bicistronic expression vector

Cloning of a bicistronic expression vector was performed as described [33]. Briefly, untagged human OTUB1 WT/N22A, FIH WT/H199A, His-OTUB1 WT/N22A and MBP-FIH WT/H199A were cloned into the transfer vector pET3a following PCR amplification. Untagged OTUB1 WT/N22A or His-OTUB1 WT/N22A was subsequently subcloned into cassette 1 of pST39, followed by subcloning of untagged FIH WT/H199A or MBP-FIH WT/H199A into cassette 4. Bacteria lysates were prepared, the protein complex purified by sequential MBP- and Ni^2+^-affinity purification and analyzed as described above.

### 2.10 Biochemical analyses of the purified stable FIH-OTUB1 complex

Equal amounts of purified FIH-OTUB1 complex or albumin (fraction V, Carl Roth GmbH + Co. KG, Karlsruhe, Germany) were either exposed to 0.1 M NaOH, 10 mM NaOH, 10 mM HCl or 1 M NH_2_OH at pH 7 or 10, or left untreated. Following incubation for 1 h at 37°C, samples were neutralized using corresponding amounts of NaOH or HCl and incubated for further 15 min at 37°C and analyzed by SDS-PAGE as described above.

### 2.11 Immunoprecipitation

Immunoprecipitation was performed as previously described [17]. Briefly, for native conditions, cells were lysed with 150 mM NaCl, 20 mM Tris-HCl (pH 7.5), 1 mM MgCl_2_, 1% Triton X-100, supplemented with protease inhibitor cocktail (Sigma-Aldrich). For denaturing conditions, cells were scraped in PBS and centrifuged for 3 min at 200 g. The cell pellet was resuspended in the same lysis buffer but supplemented with 1% SDS and 0.75 U/μl benzonase (Sigma-Aldrich), boiled for 10 min and the cellular solutes were cleared by centrifugation at 21,000 g and 4°C for 5 min. Cell lysates were incubated with anti-FLAG M2 antibody-coupled beads (Sigma-Aldrich) or anti-V5 agarose affinity gel (Sigma-Aldrich) at 4°C for 1 h. Agarose beads were washed twice with lysis buffer and twice with washing buffer (150 mM NaCl, 20 mM Tris-HCl pH 7.5, 1 mM MgCl_2_). For analysis by MS, the beads were treated as described below. For analysis by immunoblotting, the beads were resuspended in non-reducing loading buffer (50 mM Tris-HCl pH 6.8, 6% glycerol, 2% SDS, 0.01% bromophenol blue) and boiled for 5 min. 10 mM DTT was added to the supernatant and boiled for further 5 min. For endogenous FIH-specific immunoprecipitation, anti-FIH antibody or anti-β-actin control antibody was bound to protein G-sepharose (GE Healthcare) for 1 h at RT and incubated with non-denatured cell lysates over night at 4°C. Beads were washed and precipitated proteins were analyzed by immunoblotting.

### 2.12 OTUB1 deubiquitinase (DUB) assay

Purified enzymes at the indicated concentration were incubated with 600 nM K48-tetraubiquitin (K48-Ub_4_; Boston Biochem, Cambrige, MS, USA) at 37°C in the presence or absence of 25 μM UBCH5B (Enzo Life Science, Inc., Farmingdale, NY, USA). K48-Ub_4_ alone was used as negative control. The reaction was stopped by addition of 5x loading dye and samples were incubated for 20 min at RT prior to immunoblot analysis.

### 2.13 Mass spectrometry (MS) analysis of the FIH-OTUB1 HD

For analysis of the stable FIH-OTUB1 complex, immunoprecipitated proteins from HEK293 cells were separated by SDS-PAGE. Bands were cut from the Coomassie-stained gel, chopped into small pieces and washed twice with 100 mM NH_4_HCO_3_, 50% acetonitrile and once with acetonitrile. Digestion with 50 ng trypsin (sequencing grade; Promega, Madison, WI, USA) was performed in buffered conditions (10 mM Tris-HCl pH 8.2, 2 mM CaCl_2_) for 30 min at 60°C in a microwave oven (Discover System, CEM Corporation, Matthews, NC, USA). The supernatant was collected and lyophilized in a SpeedVac (Thermo Fisher Scientific). For liquid chromatography-tandem MS (LC-MS/MS) analysis, the samples were dissolved in 20 μl 0.1% formic acid and 3 μl were analyzed on a nanoAcquity ultra performance liquid chromatography (UPLC) column (Waters Corporation, Milford, MA, USA) connected to a Q Exactive mass spectrometer (Thermo Fisher Scientific) equipped with a Digital PicoView source (New Objective, Inc., Woburn, MA, USA). Peptides were trapped on a Symmetry C18 trap column (5 μm, 180 μm × 20 mm, Waters Corporation) and separated on a BEH300C18 column (1.7 μm, 75 μm × 150 m, Waters Corporation) at a flow rate of 250 nl/min using a gradient from 1% solvent B (0.1% formic acid in acetonitrile)/99% solvent A (0.1% formic acid in water) to 40% solvent B/60% solvent A within 30 min. The mass spectrometer was set to data dependent analysis, precursor scan range 350–1500 m/z, resolution 70,000, maximum injection time 100 ms, threshold 3e6. The fragment ion scan range was 200–2000 m/z, resolution 35,000, maximum injection time 120 ms, threshold 1e5. Proteins were identified using the Mascot search engine (Matrix Science; Version 2.5.1.3.). Mascot was set up to search the human SwissProt database assuming the digestion enzyme trypsin. Mascot was searched with a fragment ion mass tolerance of 0.030 Da and a parent ion tolerance of 10.0 ppm. Oxidation of methionine was specified in Mascot as a variable modification. Scaffold (Proteome Software Inc., Version 4.8.6) was used to validate MS/MS based peptide and protein identifications. Peptide identifications were accepted if they achieved a false discovery rate (FDR) of less than 0.1% by the Scaffold Local FDR algorithm. Protein identifications were accepted if they achieved an FDR of less than 1.0% and contained at least 2 identified peptides. The number of peptides was determined by the number of spectra identifying specific peptide sequences for each protein.

### 2.14 Mass spectrometry analysis of denatured and non-denatured FIH interactomes

For analysis of the stable FIH interactome by label-free quantification (LFQ), human FIH-V5 or tandem EGFP was expressed in HEK293 cells, and cells were lysed in native or denaturing conditions as described above. Following V5-specific IP, the beads were resuspended in 45 μl digestion buffer (10 mM Tris-HCl pH 8.2, 2 mM CaCl_2_) and the proteins were on-bead digested using 5 μl of 100 ng/μl trypsin in 10 mM HCl (sequencing grade; Promega) in a microwave oven for 30 min at 5 W and 60°C. The supernatants were transferred into new tubes and the beads were additionally digested for 3 h at room temperature. The beads were washed with 100 μl TFA-buffer (0.1% TFA, 10 mM Tris, 2 mM CaCl_2_) and all supernatants were combined, lyophilized, resolubilized in 25 μl of 3% acetonitrile, 0.1% formic acid spiked with iRT peptides (Biognosys AG, Schlieren, CH), centrifuged at 20,000 g for 10 min and analyzed on a Q Exactive mass spectrometer coupled to a Nano Easy 1000 liquid chromatography system (Thermo Fisher Scientific). Solvent composition was 0.1% formic acid for channel A and 0.1% formic acid in acetonitrile for channel B. For each sample, 4 μl of peptides were loaded on a commercial Acclaim PepMap Trap Column (75 μm × 20 mm, Thermo Fisher Scientific) followed by a PepMap RSLC C18 Snail Column (75 μm × 500 mm; Thermo Fisher Scientific). The peptides were eluted at a flow rate of 300 nL/min by a gradient from 5 to 22% B in 79 min, 32% B in 11 min and 95% B in 10 min. Samples were acquired in a randomized order. The mass spectrometer was operated in data-dependent mode (DDA), acquiring a full-scan MS spectra (300−1700 m/z) at a resolution of 70,000 at 200 m/z after accumulation to a target value of 3,000,000, followed by higher-energy collision dissociation (HCD) fragmentation on the twelve most intense signals per cycle. HCD spectra were acquired at a resolution of 35,000 using a normalized collision energy of 25 and a maximum injection time of 120 ms. The automatic gain control (AGC) was set to 50,000 ions. Charge state screening was enabled and singly and unassigned charge states were rejected. Only precursors with intensity above 8300 were selected for MS/MS (2% underfill ratio). Precursor masses previously selected for MS/MS measurement were excluded from further selection for 30 s, and the exclusion window was set at 10 ppm. The samples were acquired using internal lock mass calibration on *m*/*z* 371.1010 and 445.1200. The acquired raw MS data were processed by MaxQuant (Version 1.6.1) [42], followed by protein identification using the integrated Andromeda search engine [43]. Spectra were searched against a UniProt Homo Sapiens (taxonomy 9606) reference proteome (canonical version from 2016 to 12-09), concatenated to its reversed decoyed fasta database and common protein contaminants. Carbamidomethylation of cysteine was set as fixed, while methionine oxidation and N-terminal protein acetylation were set as variable modifications. MaxQuant Orbitrap default search settings were used. Enzyme specificity was set to trypsin/P. For LFQ, MaxQuant default settings were applied. In the MaxQuant experimental design template, the biological and biochemical replicates were grouped into non-adjacent fractions, to allow match-between-runs within but not between conditions. Each file was treated as a separate experiment to obtain individual quantitative values. Protein fold changes were computed based on intensity values reported in the proteinGroups.txt file. A set of functions implemented in the R package SRMService (http://github.com/protViz/SRMService [44]) was used to filter for proteins with 2 or more peptides, with quantification in at least 4 samples, and to normalize the data with a modified robust z-score transformation and to compute p-values using the *t*-test with pooled variance. The MS proteomics data were handled using the local laboratory information management system (LIMS) [45]. FIH-specific interactors had an average LFQ intensity of the four biological replicates of at least 2-fold over control (EGFP) and were statistically significantly different (p < 0.05). The obtained lists of FIH-specific interactors were analyzed for overlaps using Excel (Microsoft). Functional annotation was performed via the PANTHER database (www.pantherdb.org). For comparison of relative intensities, individual LFQ intensities were normalized to the average intensity of FIH in the samples with ectopic FIH expression.

### 2.15 Primer sequences

The designed primers for the site-directed mutagenesis of human OTUB1 were as follows (fwd, forward primer; rev, reverse primer):

S16A - fwd: 5′-accttcggagtcggcgcccagcggctcc-3′, rev: 5′-ggagccgctgggcgccgactccgaaggt-3'.
S18A - fwd: 5′-gttaacaccttcggcgtcgctgcccagcg-3′, rev: 5′-cgctgggcagcgacgccgaaggtgttaac-3'.
S16/18A - fwd: 5′-ttaacaccttcggcgtcggcgcccagcggctcc-3′, rev: 5′-ggagccgctgggcgccgacgccgaaggtgttaa-3'.
C23A - fwd: 5′-ttcatcataggccagagcgttaacaccttcggagtcgc-3′, rev: 5′-gcgactccgaaggtgttaacgctctggcctatgatgaa-3'.
C23S fwd: 5′-cataggccagagagttaacaccttcggagtcg-3′, rev: 5′-cgactccgaaggtgttaactctctggcctatg-3'.
C91A – fwd: 5′-gaaagcccgatagaaagcgttgccgtcaggcctg-3′, rev: 5′-caggcctgacggcaacgctttctatcgggctttc-3'.
C91S – fwd: 5′-aaagcccgatagaaagagttgccgtcaggcc-3′, rev: 5′-ggcctgacggcaactctttctatcgggcttt-3'.
Primers designed for the cloning of OTUB1 WT and N22A into pET3a -
fwd: 5′-actgcatatggcggcggaggaacctcagga-3′, rev: 5′-acgtggatccctatttgtagaggatatcgt-3'.
Primers designed for the cloning of FIH WT and H199A into pET3a -
fwd: 5′-acgtcatatggcggcgacagcggcgga-3′, rev: 5′-acgtggatccctagttgtatcggcccttgatca-3'.
Primers designed for the cloning of His-OTUB1 WT and N22A into pET3a - fwd 5′-acgtcatatgtcgtactaccatcaccatca-3′, rev 5′-acgtagatctctatttgtagaggatatcgt-3'.
Primers designed for the cloning of MBP into pET3a-FIH:
fwd 5′-acgtcatatgaaaatcgaagaaggtaaact-3′, rev 5′-actgcatatgggcgccctgaaaatacagg-3'.

### 2.16 Statistical analysis

For the analysis of the significance of difference between two data points, Student's t-test was applied. For comparison of more than two data points, one-way or two-way ANOVA followed by Tukey post-test was applied. P values < 0.05 were considered statistically significant.

### 2.17 Data availability

The MS proteomics data have been deposited to the ProteomeXchange Consortium via the PRIDE [46] partner repository with the dataset identifier PXD011252.

## 3 Results

### 3.1 FIH and OTUB1 form a covalently linked protein complex

**Fig. 1:**
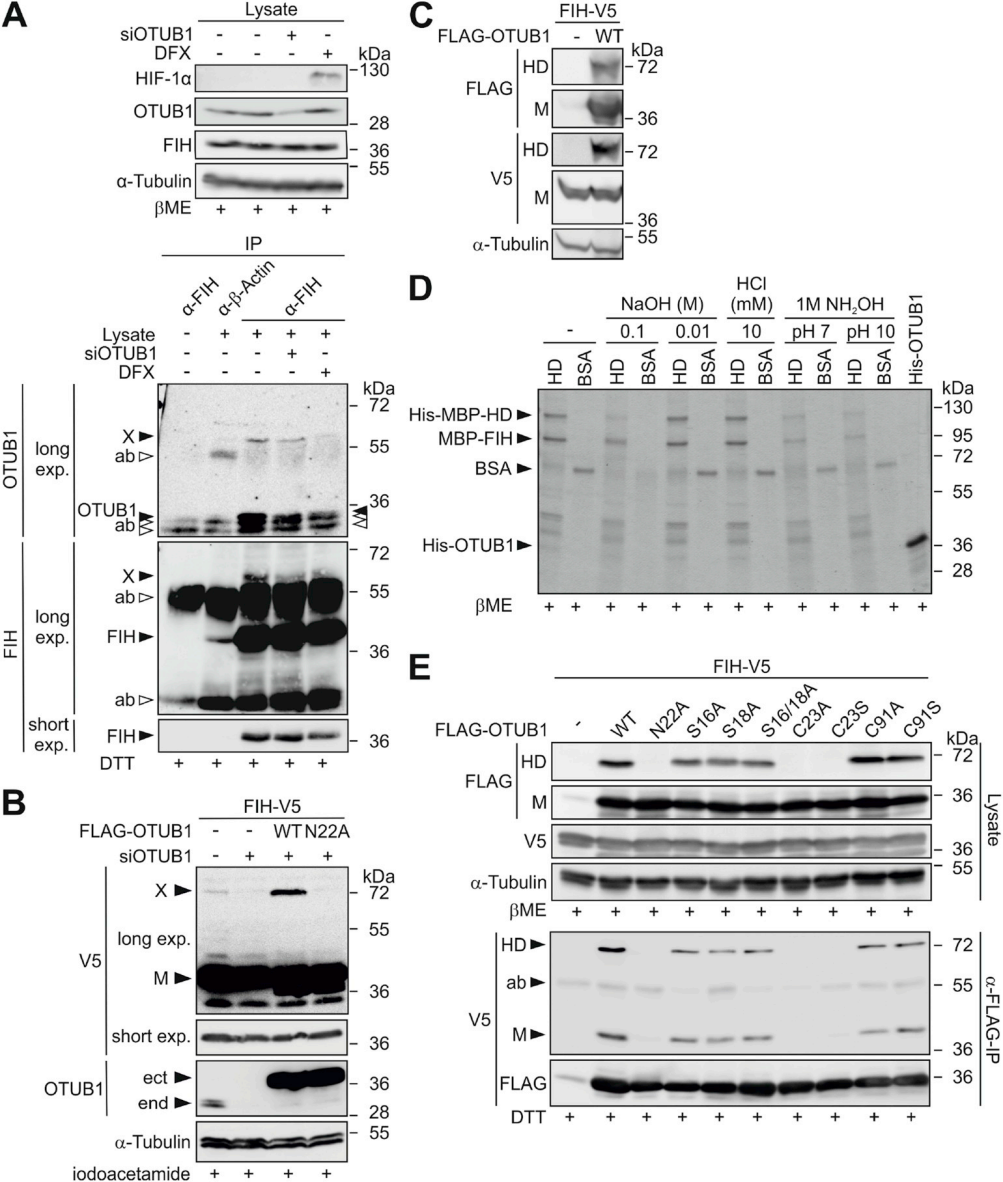
**Characterization of the FIH-OTUB1 conjugation.** (**A**) Immunoblot analysis of endogenous FIH IP detected the unexpected protein signal (“X”), which was insensitive to 858 mM β-mercaptoethanol (βME) and 10 mM DTT. The same antibody was used for the FIH IP and subsequent FIH immunoblotting and the anti-β-actin antibody was derived from the same species as the anti-FIH antibody, leading to the detection of fragments of the IP antibodies (ab; highlighted by open arrows). Black arrows highlight specific signals of the indicated proteins. (**B**) The intensity of X detected by immunoblotting of cell lysates was proportional to FIH and wildtype OTUB1 (WT) levels, as seen with ectopic FLAG-OTUB1 expression, knockdown and ectopic mutant OTUB1 (N22A) expression. Protein signal X was insensitive to 100 mM iodoacetamide, the only agent present that disrupts disulfide bonds. (**C**) Investigation of the resistance of the FIH-OTUB1 HD interaction to a chaotropic agent by urea-PAGE followed by immunoblotting. (**D**) Investigation of the effect of treatments disrupting thioesters and oxyesters using purified HD. The samples were analysed by Coomassie-stained SDS-PAGE, which included boiling in the presence of 858 mM βME during sample preparation. (**E**) Residues of the FIH interaction site of OTUB1 were mutated and their relevance for HD formation investigated by ectopic expression of the mutated proteins and anti-FLAG-IP. All samples were boiled in the presence of 858 mM βME or 10 mM DTT as indicated followed by immunoblotting exp, exposure; HD, heterodimer; M, monomer; ect, ectopic (plasmid-based) expression; end, endogenous protein. The data represent (A) two, (C, D, E (IPs)) three, (B) four or (E (lysates)) six independent experiments.

Following our previous observations of FIH-dependent hydroxylation of OTUB1 on N22 [17,18], we here investigated the interplay between FIH and OTUB1 in more detail by immunoprecipitation (IP) of endogenous FIH. Intriguingly, we detected an unexpected signal in immunoblots with antibodies derived against FIH as well as OTUB1 (Fig. 1A; marked with “X”). Protein X demonstrated a larger molecular weight than FIH or OTUB1 alone and its signal intensity was decreased following OTUB1 knockdown and abolished by iron chelation with desferrioxamine (DFX) (Fig. 1A). Protein X was also detectable using antibodies against ectopically (plasmid-based) expressed tagged FIH and OTUB1 (Figs. 1B and S1A). Protein X was subsequently investigated using ectopically expressed tagged OTUB1 and FIH to allow detailed analyses with complete control over the experimental conditions. Protein X signal intensity was proportional to OTUB1 protein levels, while mutation of the OTUB1 hydroxylation site (N22A) abrogated it (Fig. 1B). Mass spectrometry (MS) identified equimolar amounts of FIH and OTUB1 peptides in protein X (Fig. S1B). Taken together, these results are consistent with a heterodimer (HD) consisting of FIH and OTUB1. Furthermore, the FIH-OTUB1 HD was also detected in MCF7 (breast cancer) and Hep3B (liver cancer) cells (Figs. S1C and S1D), indicating that HD formation is cell type independent.

HD formation was resistant to denaturing SDS-PAGE, consistent with a possible covalent FIH-OTUB1 conjugation. This was further analyzed following the strategy of the original characterization of the covalent bond between ubiquitin (Ub) and substrate proteins [47]. The complex was resistant to chaotropic urea-PAGE (Fig. 1C). The HD was also not disrupted by high concentrations of the reducing agents β-mercaptoethanol (βME) (Fig. 1A (lysates), 1D, 1E (lysates) and S1E), iodoacetamide (Fig. 1B) and DTT (Fig. 1A (IPs) and 1E (IPs)), excluding disulfide bonds as possible connection. For the further assessment of the nature of this conjugation, the HD was purified from bacteria. The purified complex was exposed to high (0.01 and 0.1 M NaOH) and low pH (10 mM HCl) as well as to 1 M hydroxylamine (pH 7 and 10). NaOH treatment disrupts ester bonds and hydroxylamine disrupts thioester bonds at pH 7 and oxyester bonds at pH 10 [48,49]. Amide bonds are resistant to these conditions [48,49]. Low pH disrupts non-covalent bonds and esters. A specific disruption of the bond between FIH and OTUB1 in the HD should yield a decrease of HD levels with simultaneous increase in monomeric FIH and OTUB1. However, no increase in monomeric FIH or OTUB1 levels occurred when the HD decreased (Fig. 1D). In addition, when the heterodimer was reduced, the BSA control was decreased to a comparable level as well (Figs. 1D and S1F). This indicates that the observed decreases in the HD were due to general effects on protein stability and not due to specific disruption of the FIH-OTUB1 conjugation. Hence, FIH and OTUB1 are covalently attached within the HD and this covalent linkage fulfils all biochemical criteria of an amide bond.

OTUB1 N22A mutation abrogated HD formation (Fig. 1B) as well as the non-covalent interaction between FIH and OTUB1 (Fig. 1E, S2A and S2B). For a further investigation of the relevance of OTUB1 N22 for HD formation, additional mutations of OTUB1 were introduced within its FIH interaction region (S16, S18, C23) or catalytic domain (C91; leading to a catalytically inactive OTUB1 mutant [21]). Analysis by IP showed that C91 was dispensable, demonstrating that HD formation occurs independent of OTUB1 catalytic activity. The input as well as the FLAG-specific IPs showed that beside N22 also C23 mutation abrogated both the HD and the non-covalent FIH:OTUB1 interaction, while mutations of S16 and/or S18 decreased the HD and the FIH:OTUB1 interaction by roughly 50% (Fig. 1E, S2C and S2D). Overall, these results demonstrate that mutations of the OTUB1 hydroxylation site and of its FIH interaction site affect FIH-OTUB1 HD levels and that both are necessary for optimal interaction and HD formation. In addition, they indicate that the OTUB1 amino acid directly involved in the conjugation with FIH is either N22 or C23.

### 3.2 FIH-dependent FIH-OTUB1 heterodimer formation is a hypoxia-regulated mechanism

**Fig. 2:**
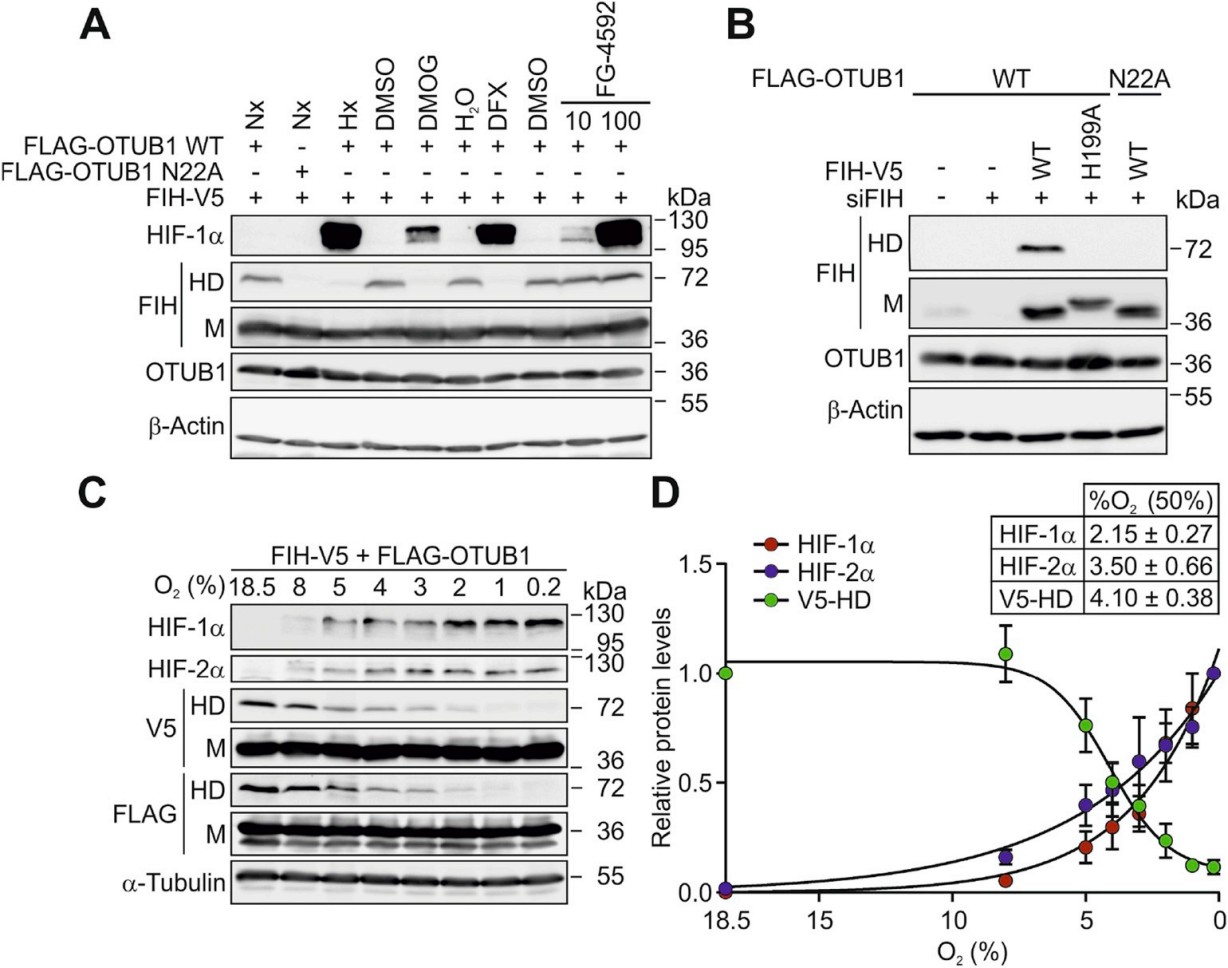
**Hypoxia sensitivity of the FIH-dependent FIH-OTUB1 heterodimer formation.** (**A**) Heterodimer (HD) formation was sensitive to hypoxia (Hx, 0.2% O_2_), DMOG and DFX, but not to the PHD-specific inhibitor FG-4592. (**B**) Immunoblotting of cell lysates with the indicated ectopic expressions. The H199A FIH mutant is catalytically inactive and was incapable of forming the HD. (**C**) Hypoxia sensitivity of HD formation in comparison with HIF-1α and HIF-2α stabilization following 24 h of incubation at the indicated O_2_ levels. (**D**) Quantification of the experiment described in (C) and calculation of the oxygen sensitivity of HD formation and HIF-1α and HIF-2α stabilization based on this quantification. Nx, normoxia; M, monomer. Data are shown as mean ± SEM from four independent experiments or are representative for (A, B) three or (C) four independent experiments.

While OTUB1 enzymatic activity was dispensable for HD formation, the OTUB1 hydroxylation site as well as the FIH interaction site were necessary. Therefore, we hypothesized that FIH catalyzes the formation of the putative covalent bond. In agreement with this hypothesis, hypoxia (0.2% O_2_), a 2-oxoglutarate (2-OG) competitor (dimethyloxalylglycine, DMOG) and the iron chelator DFX prevented HD formation (Fig. 2A). The PHD-specific inhibitor FG-4592 (roxadustat) [50] did not affect HD levels (Fig. 2A). Accordingly, knockdown of endogenous FIH with parallel expression of a catalytically inactive FIH mutant (H199A) [31] completely abolished formation of the HD (Fig. 2B). Overall, these results demonstrated that FIH enzymatic activity is required for covalent bond formation. A possibly limiting availability of FIH co-factors in cell culture affecting the FIH catalytic cycle could be excluded (Fig. S2E).

In order to investigate the sensitivity of HD formation to oxygen availability, 8 different oxygen levels were used in the range of 18.5%–0.2% O_2_. HIF-1α and HIF-2α stabilization were determined as biological readout for the obtained level of hypoxia and for the comparison with the PHD sensitivity. We observed an unusually high hypoxia sensitivity with an EC_50_ of 4.1% O_2_, which was even higher than the hypoxia sensitivity of the PHD-dependent HIF-1α and HIF-2α stabilization with EC_50_ values of 2.15% and 3.5% O_2_, respectively (Fig. 2C and D). Of note, this is in stark contrast to the known hypoxia sensitivity for FIH-dependent HIF-1α hydroxylation, which is below 1% O_2_ [51,52].

Taken together, our results demonstrate that FIH catalyses HD formation with OTUB1, which is remarkably sensitive to changes in oxygen availability within the physiologically relevant range, suggesting a function in oxygen-dependent signaling.

### 3.3 The FIH-OTUB1 heterodimer forms co-translationally and is extraordinarily stable

**Fig. 3:**
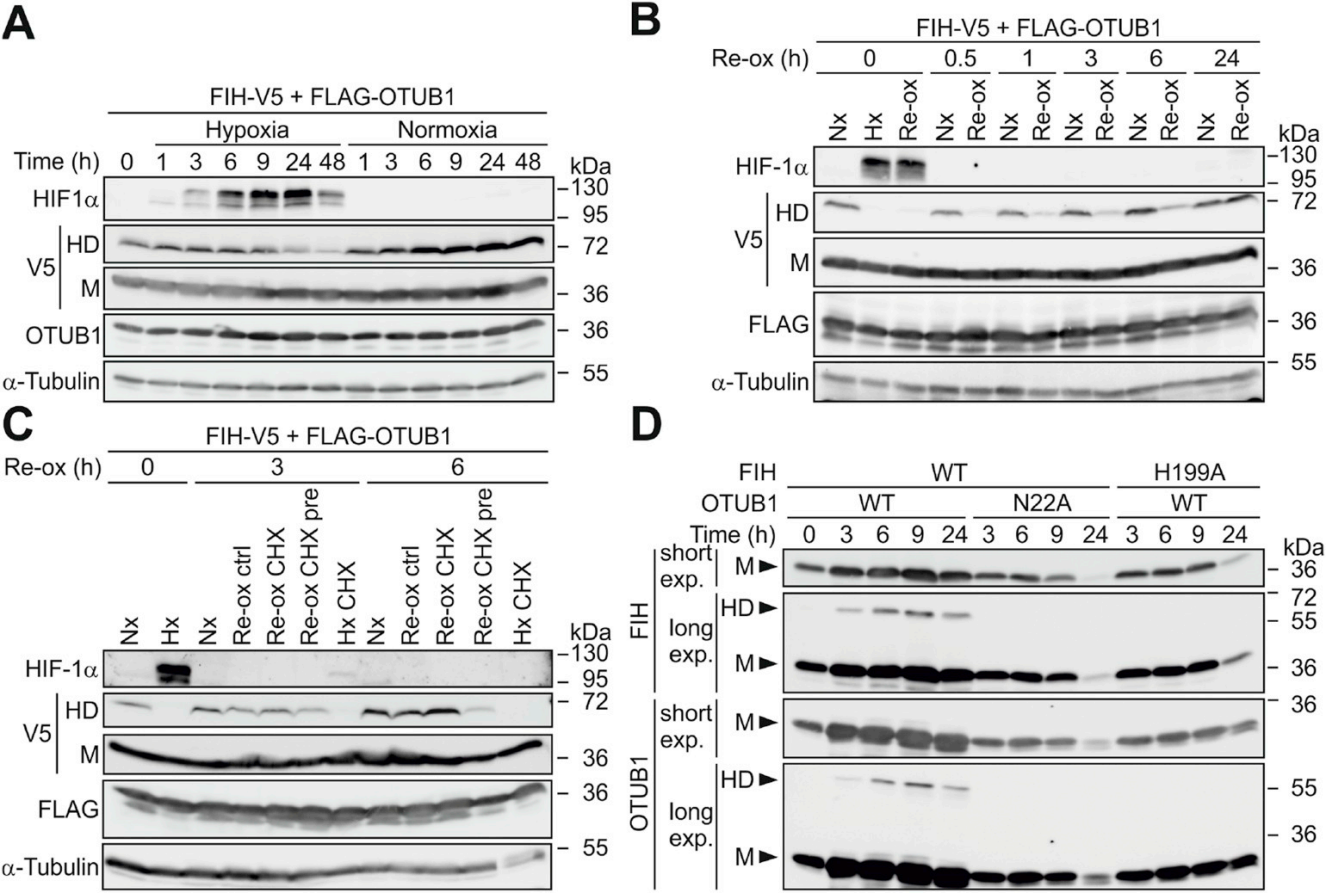
**Co-translational formation of the extraordinarily stable FIH-OTUB1 HD.** (**A**) Following ectopic expression of the indicated proteins, the FIH-OTUB1 heterodimer (HD) was allowed to form for 24 h prior to the analysis of the HD stability in hypoxia when FIH is inhibited and no additional HD can be formed. (**B**) HD formation kinetics and (**C**) HD formation during translation inhibition by cycloheximide (CHX) according to the experimental setups described in Fig. S3B. Re-Ox CHX, addition of CHX at the same time as re-oxygenation was started; Re-Ox CHX pre, pre-incubation of cells with CHX for 1 h prior to the start of re-oxygenation. (**D**) Bicistronic expression of the indicated His-OTUB1-MBP-FIH in *E.coli* followed by immunoblot analysis. Time points indicate the time after induction of protein production by addition of isopropyl-ß-D-thiogalactoside (IPTG). Nx, normoxia; M, monomer. Data are representative for three independent experiments throughout.

As the next step, the stability of the HD was analyzed *in cellulo*. First, transiently transfected cells were allowed to form the HD for 24 h with subsequent inhibition of further HD formation by exposing these cells to 0.2% O_2_. The HD showed a pronounced stability with significant decreases in HD levels after only 24 h (Figs. 3A and S3A).

For the investigation of the HD formation time, HEK293 cells were transfected with FIH and OTUB1 expressing plasmids for 5 h and subsequently incubated for 16 h in 0.2% O_2_ in order to inhibit FIH activity to express both FIH and OTUB1 without HD formation (Fig. S3B). Media was replaced with normoxic media for instantaneous re-oxygenation and the time of HD formation was analysed (Fig. S3B). HD formation was unexpectedly slow with a half-maximal level after 2.5 h, reaching a level comparable with normoxia after 8.7 h (Figs. 3B and S3C; values calculated from Fig. S3C). Re-oxygenation following hypoxia leads to active FIH within approximately 1 min [52], which can therefore not explain the slow HD formation. Hence, we assumed that a mechanism independent of FIH enzymatic activity was involved and investigated if HD formation occurred co-translationally using cycloheximide (CHX; Fig. S3B). Simultaneous addition of CHX with the start of the re-oxygenation had no effect on the formation of the HD (Fig. 3C; Re-ox ctrl vs. Re-ox CHX). However, the simultaneous start of two different treatments such as CHX and re-oxygenation can make it difficult to interpret the result due to different kinetics involved. Therefore, we also included samples in which we pre-treated the cells for 1 h with CHX prior to the start of re-oxygenation to allow for an efficient inhibition of translation at the time of re-oxygenation. In these samples, HD formation was markedly reduced after 6 h of re-oxygenation (Fig. 3C; Re-ox ctrl vs. Re-ox CHX pre), indicating that translation might be important for HD formation. Interestingly, purified FIH and OTUB1 did not form the HD under cell-free conditions (data not shown). However, a bicistronic expression vector [33] for FIH and OTUB1 (Fig. S3D), expressing both proteins in the same bacterium, resulted in HD formation which was dependent on the presence of the OTUB1 N22 hydroxylation site and on active FIH (H199A abrogated HD formation) (Fig. 3D). Co-inoculation of bacterial cultures that expressed either FIH or OTUB1 did not lead to detectable HD formation (Fig. S3E). Taken together, these results strongly suggest that HD formation occurs co-translationally.

### 3.4 The FIH-OTUB1 HD is part of a native FIH:FIH-OTUB1 heterotrimer

**Fig. 4:**
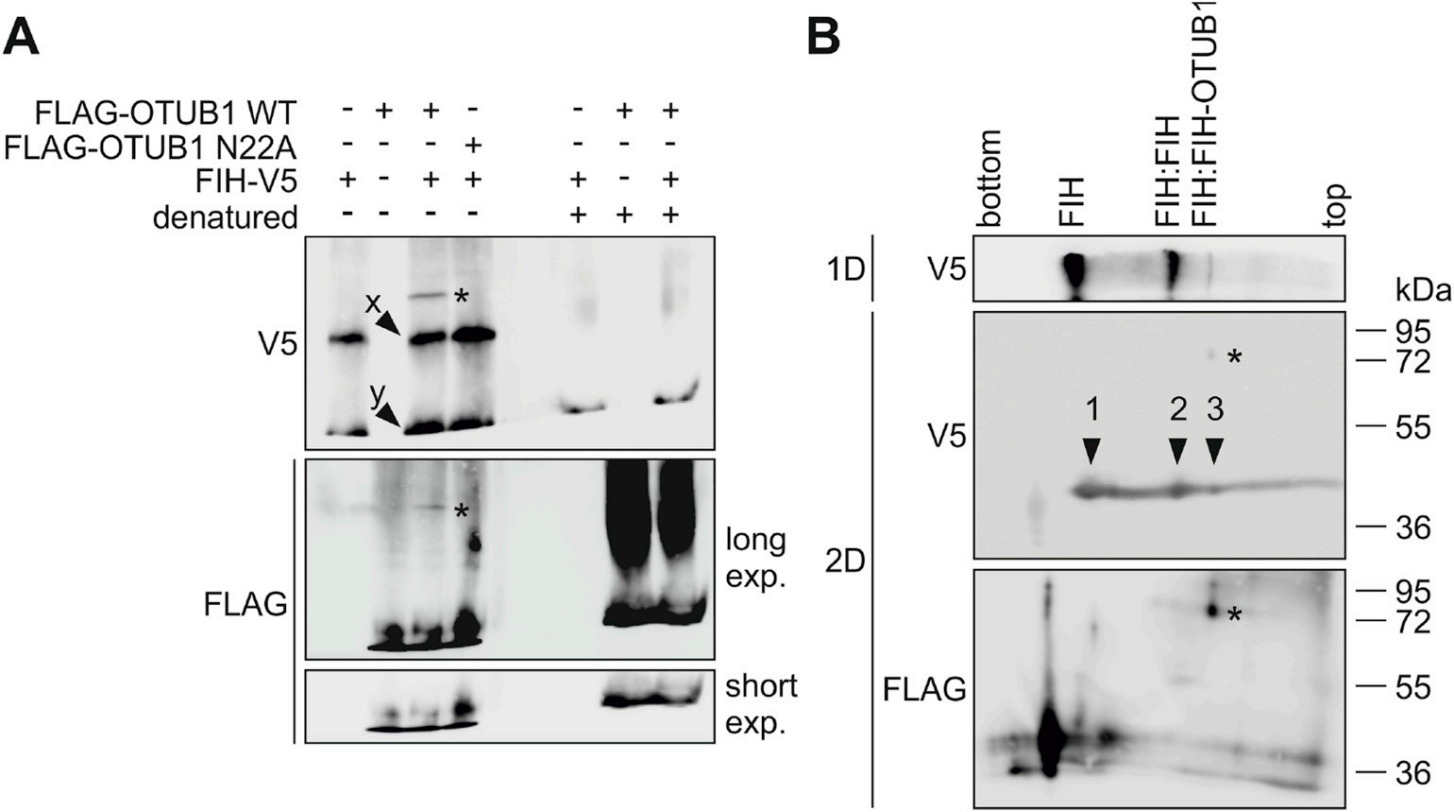
**FIH:FIH-OTUB1 heterotrimer formation.** Analysis of the composition of the covalently linked FIH-OTUB1 complex by (**A**) blue native-PAGE (first dimension, 1D) following ectopic expression in HEK293 cells and by (**B**) SDS-PAGE as second dimension (2D). *, FIH-OTUB1 heterodimer; x, FIH homodimer; y, FIH monomer; arrows 1–3, monomeric FIH originating from FIH monomers [1], FIH:FIH homodimers [2] or the FIH:FIH-OTUB1 heterotrimer [3]. Data are representative for three independent experiments throughout.

The active form of FIH is a non-covalent FIH:FIH homodimer [53]. Therefore, we sought to investigate, if both FIH proteins of the homodimer form a covalent bond with OTUB1 and if HD formation interferes with the interaction of the FIH proteins within the FIH homodimer. Following native gel electrophoresis, ectopic FIH-V5 expression alone showed two bands in the immunoblot, which corresponded to monomeric FIH and homodimeric FIH, as homodimeric FIH was not detectable anymore after denaturation of the same sample (Fig. 4A). With ectopic FIH-V5 and FLAG-OTUB1 co-expression, a complex was detectable that was composed of FIH and OTUB1 (detected with both antibodies) and moved slower in the electric field than the FIH homodimer (Fig. 4A). Next, the composition of this complex was analyzed in a second denaturing dimension following native gel electrophoresis. Alongside the HD, a further signal was observed in the anti-V5 immunoblot at the same molecular weight as the V5 signal obtained from FIH-V5 expression alone (Fig. 4B). This revealed that the native complex contained an additional, non-covalently bound FIH protein. Non-covalently interacting (monomeric) OTUB1 was not detected within the complex (Fig. 4B). In summary, only covalently linked OTUB1 was present in the complex combined with a covalently linked FIH and a second, non-covalently interacting FIH. Hence, *in cellulo* a FIH:FIH-OTUB1 heterotrimer (HT) is formed.

### 3.5 Covalent complex formation with FIH regulates OTUB1 enzymatic activity

**Fig. 5:**
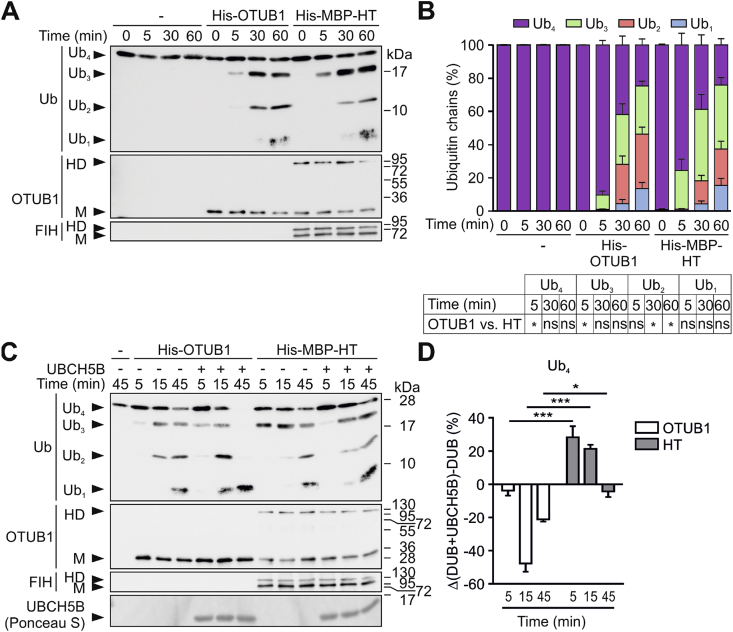
**UBCH5B-dependent regulation of OTUB1 DUB activity in the FIH:FIH-OTUB1 heterotrimer.** (**A-D**) Comparison of the OTUB1 enzymatic activity in purified monomeric OTUB1 and in the FIH:FIH-OTUB1 heterotrimer (HT) in (**A**, **B**) the absence or (**C**, **D**) presence of the E2 protein UBCH5B using (**A**, **C**) a DUB assay and (**B**, **D**) quantification. (**D**) The relative levels of Ub_4_ chains were quantified in each sample of the experiment described in (C). Quantified Ub_4_ chain amounts in the samples containing His-OTUB1 alone were subtracted from the quantified Ub_4_ chain amounts in samples with His-OTUB1 + UBCH5B (clear bars). The same analysis was carried out for the His-MBP-HT: quantified amounts of Ub_4_ chains in samples with His-MBP-HT alone were subtracted from the quantified amounts in the samples containing His-MBP-HT + UBCH5B (grey bars). DUB, indicates the deubiquitinases OTUB1 or HT, respectively. HD, heterodimer; M, monomer, Ub_n_, K48-linked ubiquitin chains with n number of Ub proteins; ns, not significant. Data are shown as mean + SEM from three independent experiments or are representative for three independent experiments. *, p < 0.05; **, p < 0.01; ***, p < 0.001 by two-way ANOVA followed by Tukey post-test.

To assess possible functional consequences of HT formation for OTUB1 enzymatic activity (hydrolysis of K48-linked Ub chains), monomeric free OTUB1 and the native HT were purified from bacterial lysates (Figs. S4A and S4B). To account for a small contamination of the HT by monomeric OTUB1, indicating that non-covalently bound OTUB1 was co-purified, molar concentrations of the control (monomeric OTUB1) were matched with the contamination. Within the HT sample, there was a significant increase in cleavage of K48-Ub chains in comparison to monomeric OTUB1 following 5 min of incubation, as shown by significant decreases in Ub_4_ chains paralleled by significant increases in Ub_3_ chains (Fig. 5A and B). This demonstrated a higher deubiquitinase activity within the HT sample over monomeric OTUB1, which could only be derived from the HT itself. Hence, OTUB1 enzymatic activity was maintained in the HT. Interestingly, at later time points this activity was decreased in comparison to monomeric OTUB1 (Fig. 5A and B).

Interaction of the OTUB1 N-terminus with uncharged E2s (such as UBCH5B) increases OTUB1 activity towards K48-ubiquitin chains by stabilizing the structure of an OTUB1 Ub-binding site [26]. Because the OTUB1 N-terminus also contains the FIH interaction site, we investigated if the stimulating effect of UBCH5B on OTUB1 enzymatic activity was affected by HT formation. The activity of purified monomeric OTUB1 was strongly increased in the presence of UBCH5B, as demonstrated by a faster turnover of K48-linked Ub_4_ into smaller Ub chains when UBCH5B was present (Fig. 5C and D and Fig. S4C). In contrast, HT-dependent cleavage of Ub_4_ chains was reduced in the presence of UBCH5B in comparison to HT alone, as shown by higher residual levels of Ub_4_ chains in samples containing both the HT and UBCH5B compared to samples containing the HT alone (Fig. 5C and D and Fig. S4C). This demonstrated that UBCH5B had the opposite effect on OTUB1 activity when OTUB1 was bound by FIH (forming the HT) than on monomeric OTUB1.

These results show that OTUB1 maintains its enzymatic activity within the heterotrimeric complex with FIH, but the important regulation of its activity by the E2 enzyme UBCH5B is inverted, demonstrating a functional effect of FIH:FIH-OTUB1 HT formation.

### 3.6 FIH forms denaturation resistant complexes with a specific subset of its interactome

**Fig. 6:**
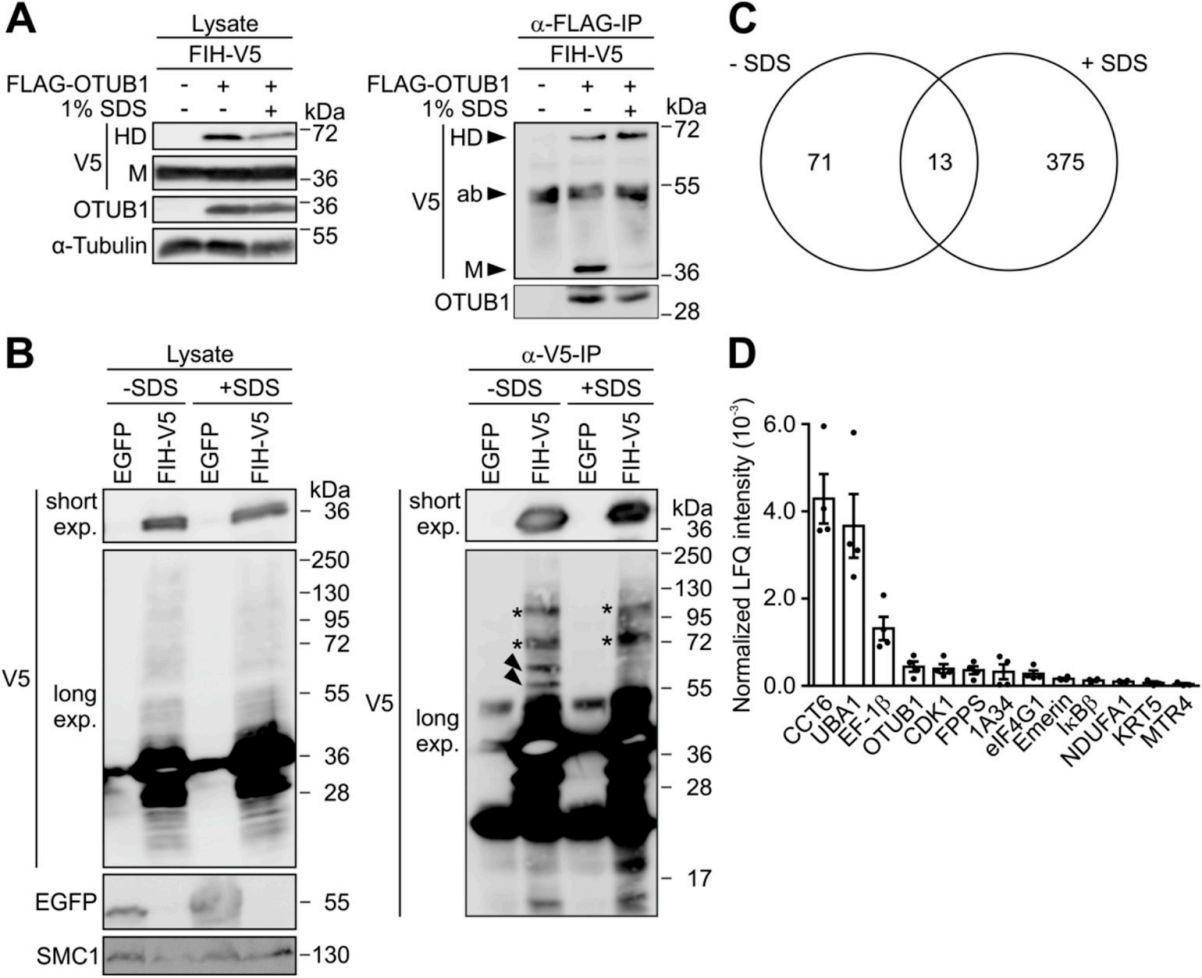
**Denaturing condition-resistant FIH interactome composition.** (**A**) Following ectopic expression of the indicated proteins, HEK293 cells were lysed under native or denaturing (boiling in 1% SDS) conditions followed by anti-FLAG IP and immunoblot analysis. HD, heterodimer; M, monomer; ab, antibody. (**B**) Following ectopic expression of either FIH-V5 or tandem EGFP, HEK293 cells were lysed as described in (A) followed by anti-V5 IP and immunoblot analysis. Arrow heads indicate protein signals that were only detected following IP from native samples (-SDS). *, protein signals detected in IPs from both conditions (native and denatured lysates); exp, exposure. (**C**) Following the same sample preparation as in (B), the samples were analysed by MS. Venn diagram displaying the overlap between FIH interactors under native (-SDS) and denaturing (+SDS) conditions. (**D**) Rank order of the 13 proteins interacting with FIH under both conditions shown in (C), according to the relative label free quantification (LFQ) intensity following IP in the presence of SDS and normalized to FIH pull-down. Data are shown as mean ± SEM. HD, heterodimer; M, monomer; ab, antibody; exp, exposure. Data are shown as mean ± SEM from (C, D) four biological replicates or are representative for (A) three or (B) one independent experiment. (B–D) Samples were processed in parallel and only differ in the analysis (immunoblot or MS).

During our analyses, we observed further higher molecular weight bands in addition to the HD that were also detected with an antibody against FIH (Fig. S5A). Intriguingly, these bands disappeared when FIH activity was inhibited (Fig. S5A). This indicated that FIH-dependent covalent bond formation was not restricted to OTUB1. For the investigation of such potential further covalent complexes formed by FIH, we utilized an assay previously described for the discrimination of covalent and non-covalent ubiquitin interactions [54]. In this assay, the FIH-OTUB1 HD was pulled down under denaturing conditions without non-covalently interacting FIH (Fig. 6A). When FIH-V5 was expressed alone, the same approach showed several high molecular weight complexes, of which some were maintained under denaturing conditions (”+SDS”) (Fig. 6B). This further indicated that a subset of the FIH interactome forms covalent complexes with FIH similar to OTUB1. MS identified 71 proteins that interacted with FIH following native lysis (”- SDS”), while 375 proteins were observed following IP from denatured cell lysates (Fig. 6C). The higher number of co-precipitants in the IP from denatured cell lysates was surprising, but denaturing lysis will lead to the extraction of more proteins, which could explain the difference in the number of detected proteins. Thirteen FIH interactors were present under denaturing as well as native conditions, including OTUB1 (Fig. 6C and D and Fig. S5B). The interactomes covered a broad spectrum of different biological processes (Fig. S5C). Among the 12 novel candidates for covalent complex formation, the previously described FIH interactors IκBβ and CDK1 were present [13,17]. These results demonstrate that FIH forms stable complexes with a subset of its interactome.

## 4 Discussion

The formation of protein complexes is the basis for cellular processes and functions [55]. Hence, the understanding of protein complex formation is fundamental for our understanding of health and disease. Cellular oxygen sensing is vital for cells in order to be able to monitor oxygen availability in their local microenvironment and to adjust to changes accordingly [3]. In this study, we provide insights into a previously unknown cross-talk between FIH and OTUB1 through amide bond formation catalysed by FIH, with unprecedented oxygen sensitivity and with functional relevance for OTUB1, regulating its K48-Ub chain cleavage activity. The covalent FIH-OTUB1 HD formation may represent an alternative molecular mechanism for the cellular adaptation to oxygen changes over longer time periods, linking oxygen sensing and deubiquitinase activity. Furthermore, we provided results indicating that FIH-dependent covalent bond formation is not exclusive for OTUB1.

The covalent bond of the FIH-OTUB1 HD fulfils all biochemical criteria of an amide bond [[47], [48], [49],^56^,^57^]. FIH enzymatic activity was necessary for HD formation and hence FIH appears to have amide synthase activity. This activity required the same co-factors and co-substrates as FIH-dependent hydroxylation. But in contrast to amide bond formation in the ubiquitin system, FIH is not known to utilize ATP. Furthermore, the catalyzing protein (FIH) attaches itself instead of a third moiety, such as an ubiquitin or a hydroxyl group. Hence, the proposed FIH amide synthase activity on OTUB1 would be based on an unprecedented molecular mechanism.

FIH can oxidize hydrophilic, hydrophobic, acidic, basic, polar and neutral amino acid side chains and FIH may catalyze the formation of β–oxo-histidine and dehydrohistidine [[58], [59], [60]]. This demonstrates that FIH catalytic activity is highly promiscuous and that FIH may be capable of catalyzing more than asparagine hydroxylation. In our experiments, point mutations of OTUB1 indicated that N22 and C23 are necessary for HD formation. Cysteines such as C23 can form disulfide bonds or thioesters, but both types of covalent bonds were excluded by our analyses. N22 can be hydroxylated by FIH [18], demonstrating that N22 is accessible for FIH catalytic activity. Therefore, N22 is likely the OTUB1 amino acid that is involved in the covalent bond formation. However, peptides corresponding to the suspected regions of FIH-OTUB1 HD formation could not be detected by MS, which is likely due to the unknown molecular weight of these unique peptides, and excludes a direct examination of the nature of the FIH-OTUB1 bond.

Interestingly, asparagine can non-enzymatically form succinimide intermediates, which lead to amide bond formations with lysine residues during aging [61]. Furthermore, in specific bacterial proteins asparagine can auto-catalytically form succinimide intermediates that lead to amide bond formation with lysyl residues, provided that an additional glutamate or aspartate is present within a hydrophobic pocket [62]. FIH contains an aspartate (D201; iron coordination) and a lysyl residue (K214; 2-OG coordination) within its active center [63,64]. Unfortunately, the involvement of K214 in covalent bond formation cannot be assessed since the enzymatic activity of FIH is likely lost following K214 mutation.

FIH-dependent asparagine hydroxylation of HIF still occurs at lower oxygen levels than PHD dependent prolyl hydroxylation [52,65]. Here, we report an even higher oxygen sensitivity for FIH-mediated HD formation than for PHD-mediated HIFα destabilization. The half-maximal oxygen concentration (gas phase) for HD formation was determined as 4.1% O_2_, which is in stark contrast to the previously determined sensitivity for FIH-dependent HIFα hydroxylation, being below 1% O_2_ [51,52]. Interestingly, the oxygen sensitivity of FIH-dependent hydroxylation depends on the used substrate and its length [66,67]. Therefore, it is likely that the here observed unprecedentedly high oxygen sensitivity of FIH is encoded within the interacting peptide of the specific substrate.

Functionally, OTUB1 maintained enzymatic activity within the FIH:FIH-OTUB1 HT, while the regulation of its activity by UBCH5B was affected. OTUB1 enzymatic activity is regulated by E2 enzymes dependent on the presence of free mono-Ub and whether the E2 is charged with a covalently attached Ub. Mono-Ub in combination with Ub-charged E2 enzymes inhibits OTUB1 enzymatic activity due to interaction of the Ub of the charged E2 enzyme with an Ub binding site at the OTUB1 N-terminus and the interaction of the free mono-Ub with a second Ub binding site, preventing OTUB1 from binding its substrate (K48-linked Ub chains) [23,26,27]. Uncharged E2 enzymes in turn stimulate OTUB1 activity by stabilizing the structure of the N-terminal Ub-binding site that is disordered in the apoenzyme [21,26,27]. When we assessed if OTUB1 DUB activity was preserved within the HT (in the absence of UBCH5B), we observed an initial increase of OTUB1 activity at 5 min, which decreased in comparison to non-complexed OTUB1 at later time points, coinciding with an increased release of mono-Ub. An E2 was not present, but FIH might mimic the effect of a charged E2 enzyme within the HT, as it also binds to the OTUB1 N-terminus. The stimulation of OTUB1 activity by uncharged UBCH5B was inverted when OTUB1 was complexed by FIH. This effect was again comparable to the regulation of OTUB1 activity by a charged E2 enzyme, although this time in the absence of free Ub. Overall, it seems likely that the functional regulation of OTUB1 by covalently bound FIH:FIH is due to its localization and its resemblance to an interacting charged E2 enzyme.

We observed that the formation of the FIH-OTUB1 heterodimer is slow (within the range of several hours) combined with slow degradation kinetics (up to 24 h). This is in stark contrast to the fast HIF-1α stabilization and degradation kinetics (seconds to minutes) [68]. The fast HIF-1α kinetics is crucial for its role as the main transcription factor for the cellular adaptation especially to acute changes in oxygen levels. The observed slow formation and degradation kinetics of the FIH-OTUB1 HD will make it insensitive to brief fluctuations of oxygen levels (minutes to possibly a few hours). Hence, the FIH-OTUB1 complex is likely not involved in acute but rather in chronic cellular adaptations to hypoxia, providing a further set point for cellular oxygen availability besides HIFα.

Interestingly, the monomeric prolyl-3-hydroxylase 2-OG and Fe(II)-dependent oxygenase domain-containing protein 1 (OGFOD1) has been shown to form an OGFOD1 activity-dependent SDS-PAGE resistant complex with its substrate ribosomal protein S23 (RPS23) [69,70]. However, the oxygen sensitivity of the complex formation, a possible functional consequence or the nature of the interaction remained unclear. Of note, a point mutation in RPS23 that impairs its hydroxylation and stable complex formation with OGFOD1 has recently been linked to ribosomopathy in humans [71], indicating that covalent HD formation of hydroxylases with their substrates may be involved in human diseases.

## Declarations of interest

None.
