# Supplementary material for: Oxygen-dependent bond formation with FIH regulates the activity of the client protein OTUB1
Source: Redox Biol. 2019 Jul 2;26:101265. doi: 10.1016/j.redox.2019.101265 (PMC6624438; doi:10.1016/j.redox.2019.101265)
Supplement: Multimedia component 2 [file mmc2.docx]

**Supplementary Data**

**Oxygen-dependent bond formation with FIH regulates the activity of the client protein OTUB1**

**Christina Pickel, Julia Günter, Amalia Ruiz-Serrano, Patrick Spielmann, Jacqueline-Alba Fabrizio, Witold Wolski, Daniel J. Peet, Roland H. Wenger, and Carsten C. Scholz**


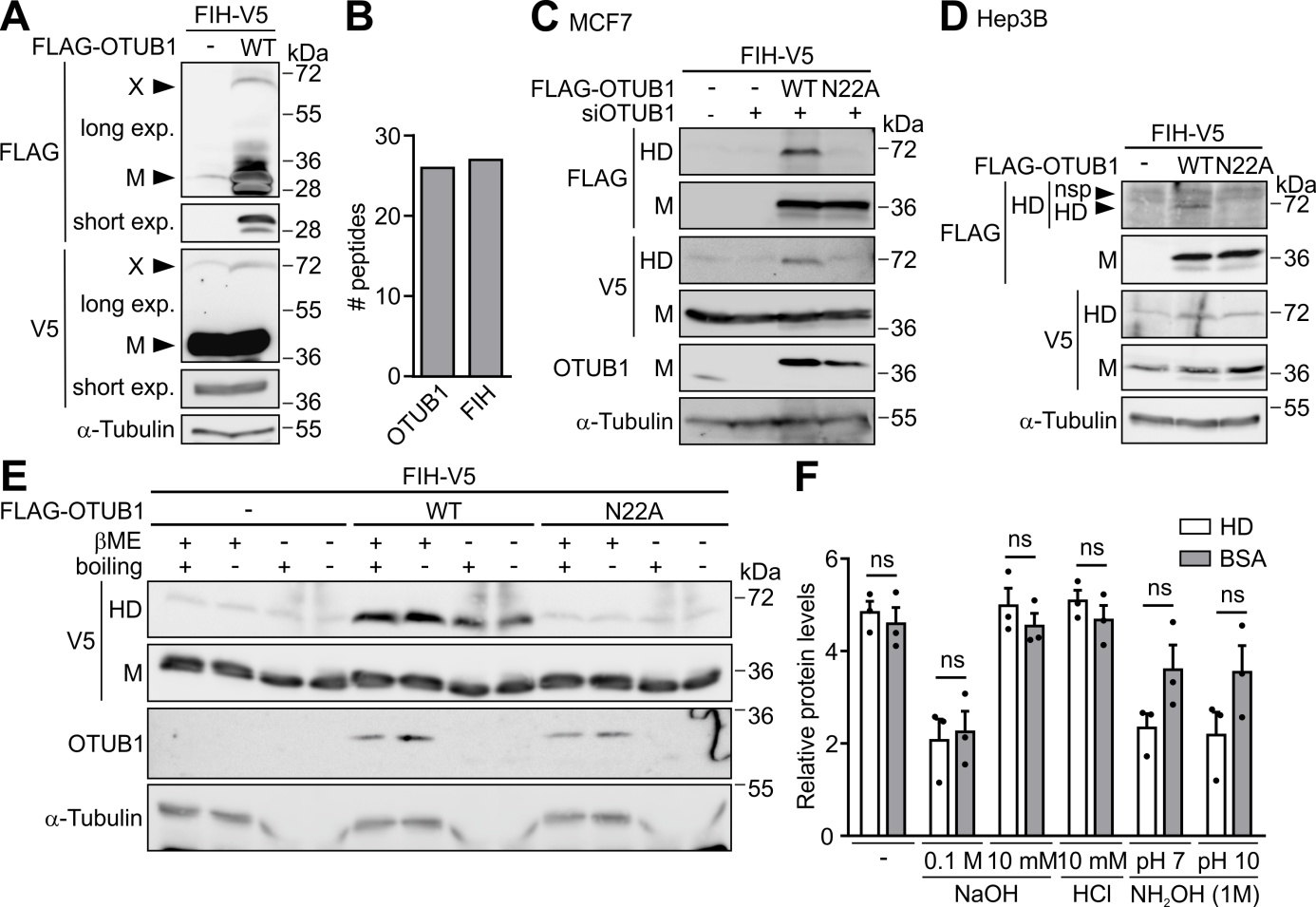


**Fig S1. Cell-type independent covalent FIH-OTUB1 HD formation.** (**A**) Ectopic expression of FLAG-OTUB1 in combination with FIH-V5 led to the detection of protein signal “X” at 72 kDa with both anti-FLAG and anti-V5 antibodies. (**B**) MS analysis of protein X excised from a SDS-PAGE gel following IP from HEK293 cell lysates demonstrated that protein X contained both OTUB1 and FIH. (**C**, **D**) Detection of the heterodimer (HD) by immunoblotting in MCF7 breast adenocarcinoma (C) and Hep3B liver carcinoma cell lysates (D) with the indicated plasmid-dependent expressions and knockdowns. (**E**) Immunoblot analysis of HEK293 lysates with the indicated ectopic expression in the presence or absence of 858 mM β-mercaptoethanol (βME) and boiling. (**F**) Quantification of the experiment described in Fig 1D. M, monomer; ab, antibody; nsp, non-specific. Data are shown as mean + SEM from (F) three independent experiments or are representative for (B) one or (A, C-F) three independent experiments. Statistical analysis by two-way ANOVA followed by Tukey post-test (ns, not significant).


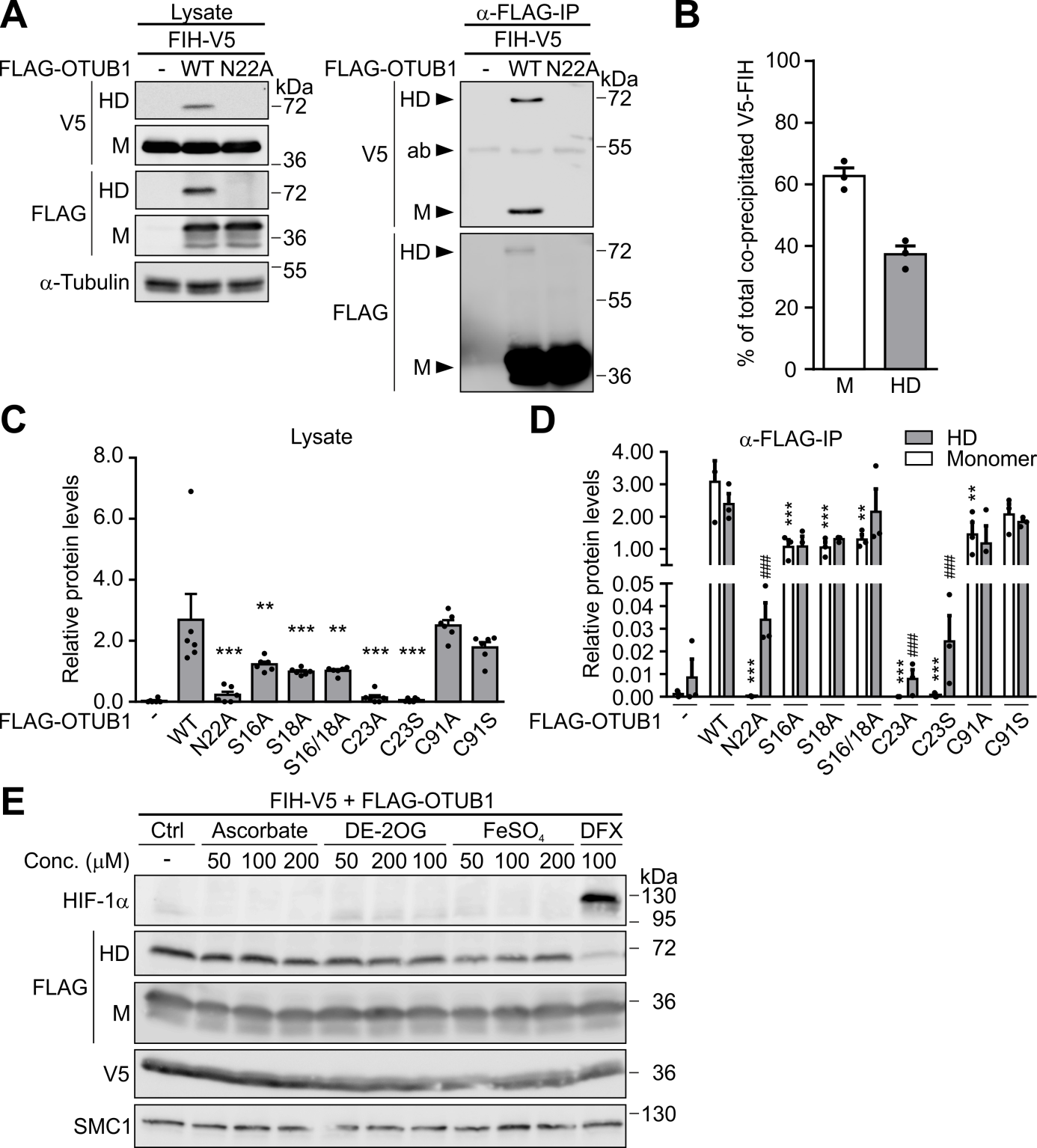


**Fig S2. Formation of the FIH-OTUB1 HD depends on the OTUB1 hydroxylation site and FIH activity.** (**A**) Immunoblotting following anti-FLAG IP of HEK293 lysates containing the indicated ectopically expressed proteins. (**B**) Quantification of monomeric FIH (M) and of the FIH-OTUB1 HD of the experiments shown in (A). (**C**, **D**) Quantification of the experiments shown in Fig 1E. (**E**) Immunoblot analysis of HEK293 cells following the addition of FIH co-factors/substrates or the iron chelator DFX. HD, heterodimer; M, monomer; ab, antibody. Data are shown as mean + SEM from (B, D) three or (C) six independent experiments or are representative of (A, E) three independent experiments. **, p<0.01; ***^/###^, p<0.001 by one-way ANOVA followed by Tukey post-test. *, statistically significant compared with HD WT; #, statistically significant compared with M WT.


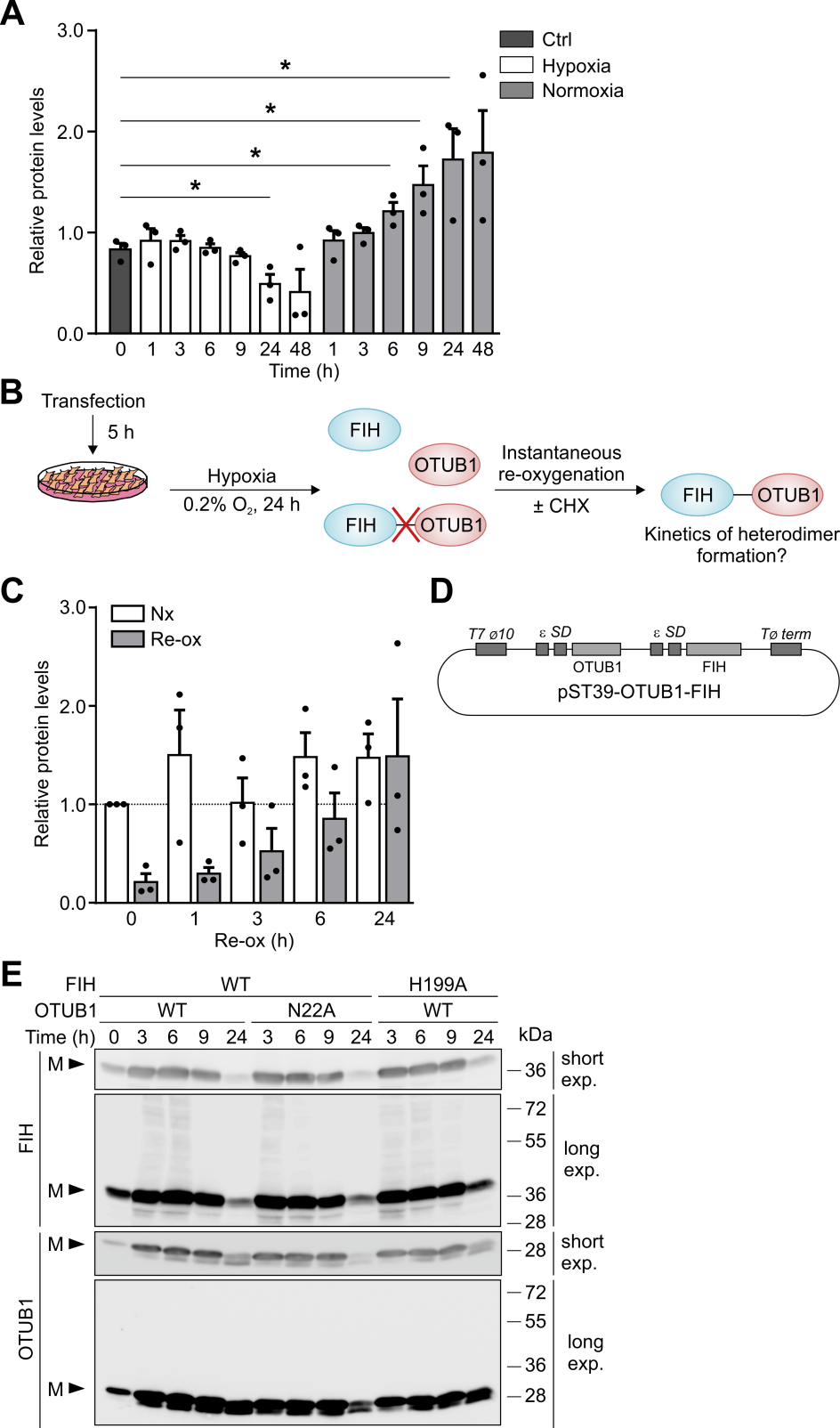


**Fig S3. FIH-OTUB1 HD stability and formation kinetics.** (**A**) Quantification of the experiment depicted in Fig 3A. (**B**) Illustration of the experimental procedures performed in Fig 3B and 3C. HEK293 cells were transiently co-transfected with FIH-V5 and FLAG-OTUB1 WT, incubated in hypoxia (0.2% O_2_) for 24 h and instantaneously re-oxygenated in the presence or absence of 400 µM cycloheximide (CHX). (**C**) Quantification of the experiment depicted in Fig 3B. (**D**) Plasmid map of the bacterial bicistronic expression vector pST39-His-OTUB1-MBP-FIH. T7 Ø10, T7 promotor; T7 term, T7 terminator; ε, translational enhancer; SD, Shine-Dalgarno sequence. (**E**) Immunoblot analysis of co-inoculated bacterial cultures expressing either OTUB1 or FIH. Bacteria were transformed with either OTUB1 or FIH expression plasmids, cultivated separately and then mixed in a 1:1 ratio followed by induction of the indicated protein expression. HD, heterodimer; M, monomer; exp., exposure. Data are shown as mean + SEM from three independent experiments or are representative for (E) two independent experiments.


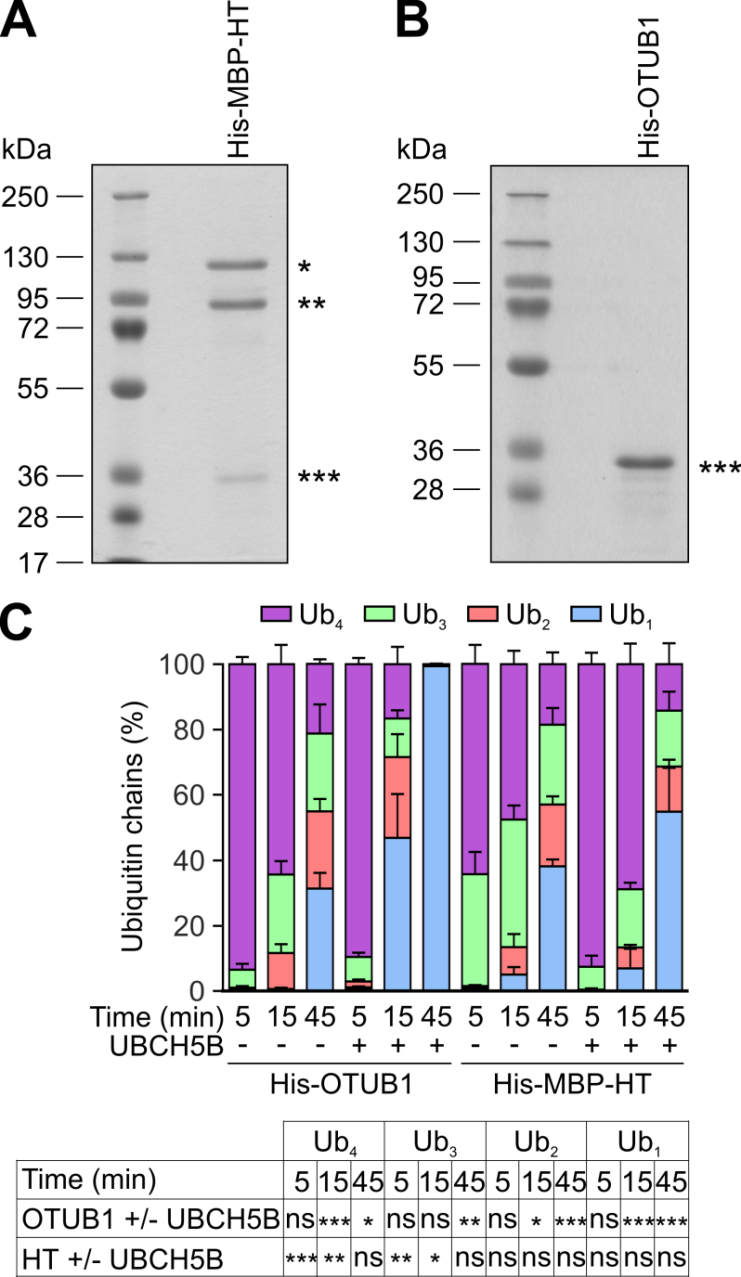


**Fig S4. Quality control of purified FIH:FIH-OTUB1 HT and His-tagged OTUB1 and quantification of *in vitro* DUB activity assay.** (**A**) His-OTUB1-MBP-FIH:FIH-MBP heterotrimer (His-MBP-HT) was purified from *E.coli* by sequential MBP- and Ni^2+^-affinity purification and analyzed by SDS-PAGE and colloidal Coomassie staining. (**B**) His-OTUB1 was purified from *E.coli* by Ni^2+^-affinity purification and analyzed by SDS-PAGE and colloidal Coomassie staining. *, FIH-OTUB1 heterodimer (predicted MW: 117.82 kDa); **, monomeric MBP-FIH originating from the native protein complex (predicted MW: 83.78 kDa); ***, monomeric His-OTUB1 (predicted MW: 34.04 kDa). (**C**) Quantification of each single Ub chain of the experiment depicted in Fig. 5C. Data are shown as mean + SEM from three independent experiments. *, p<0.05; **, p<0.01; ***, p<0.001 by two-way ANOVA followed by Tukey post-test.


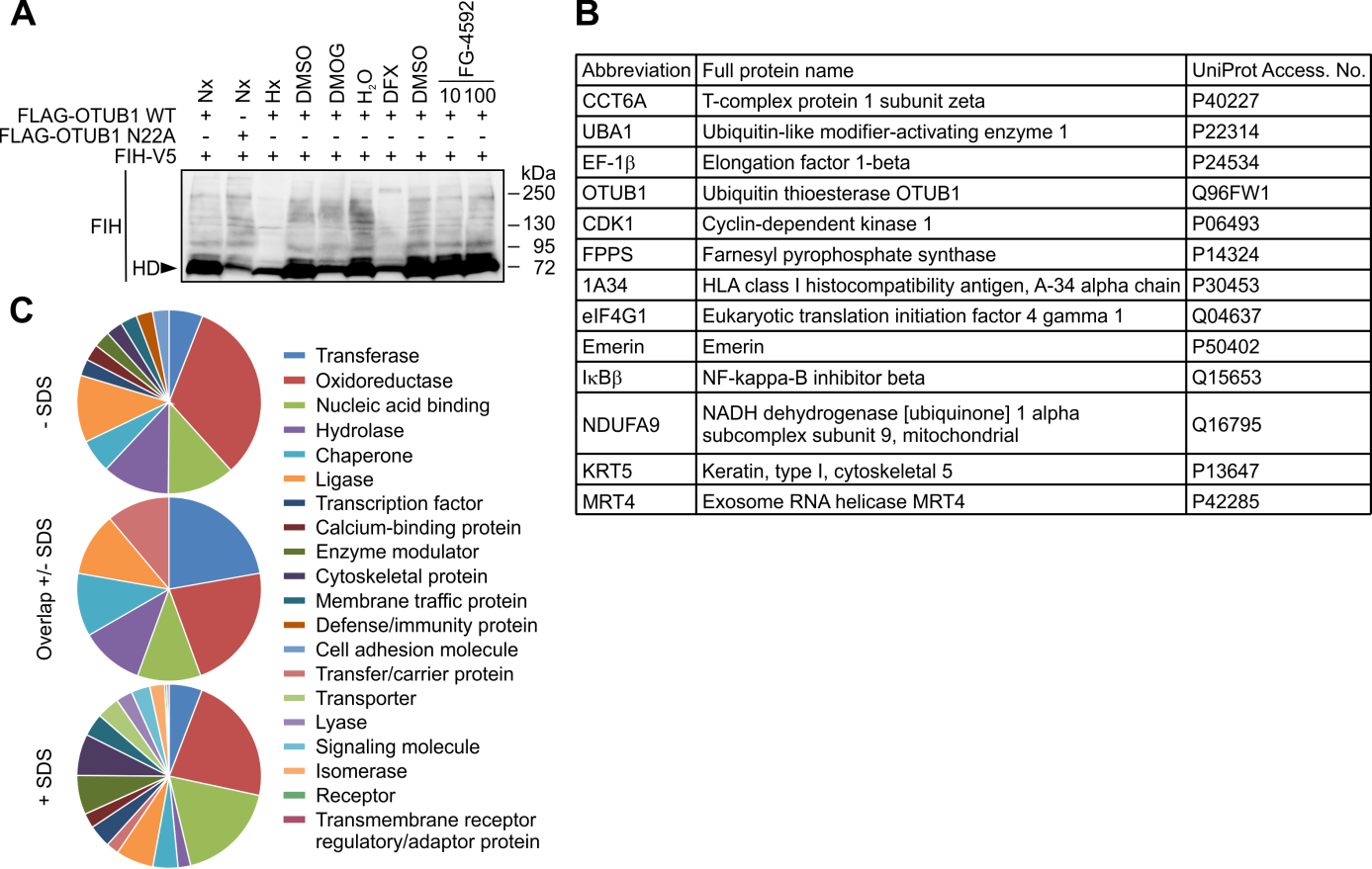


**Fig S5. FIH interactome under native and denaturing conditions.** (**A**) Longer exposure of the FIH immunoblot shown in Fig 2A. (**B**) Protein abbreviations, full names and uniprot.org identifiers of proteins detected as FIH interactors under both lysis conditions. (**C**) Proteins shown in Fig 6C were analyzed and categorized for protein class using the Panther classification system ([www.pantherdb.org](http://www.pantherdb.org)). HD, heterodimer. Data were analyzed from (B, C) four biological replicates or are representative for (A) three independent experiments.
